# Supplementary material for: Presence of phage-plasmids in multiple serovars of Salmonella enterica
Source: Microb Genom. 2024 May 8;10(5):001247. doi: 10.1099/mgen.0.001247 (PMC11165635; doi:10.1099/mgen.0.001247)
Supplement: Uncited Fig. S1. [file mgen-10-01247-s001.pdf]

Presence of phage-plasmids in multiple serovars of *Salmonella enterica*

Satheesh Nair[1], Clare R Barker[1,2], Matthew Bird[1,3], David R Greig[1,4,5], Caitlin Collins[1,2,6], Anaïs Painset[1], Marie Chattaway[1,2], Derek Pickard[7], Lesley Larkin[1], Saheer Gharbia[1,2,3], Xavier Didelot[2,4,8], Paolo Ribeca[1,2,4,9]

1) UK Health Security Agency, London, UK

2) NIHR Health Protection Research Unit in Genomics and Enabling Data, University of Warwick, UK

3) NIHR Health Protection Research Unit in Healthcare Associated Infections and Antimicrobial Resistance, University of Oxford, UK

4) NIHR Health Protection Research Unit in Gastrointestinal Infections, University of Liverpool, UK

5) Division of Infection and Immunity, The Roslin Institute and Royal (Dick) School of Veterinary Studies, University of Edinburgh, UK

6) Department of Veterinary Medicine, University of Cambridge, UK

7) The Cambridge Institute for Therapeutic Immunology and Infectious Disease (CITIID), University of Cambridge, UK

8) School of Public Health and Department of Statistics, University of Warwick, UK

9) Biomathematics and Statistics Scotland, The James Hutton Institute, UK

Corresponding authors: paolo.ribeca@ukhsa.gov.uk & satheesh.nair@ukhsa.gov.uk

Keywords: *Salmonella*, phage-plasmid, antimicrobial resistance, genomic surveillance

Supplementary Figures

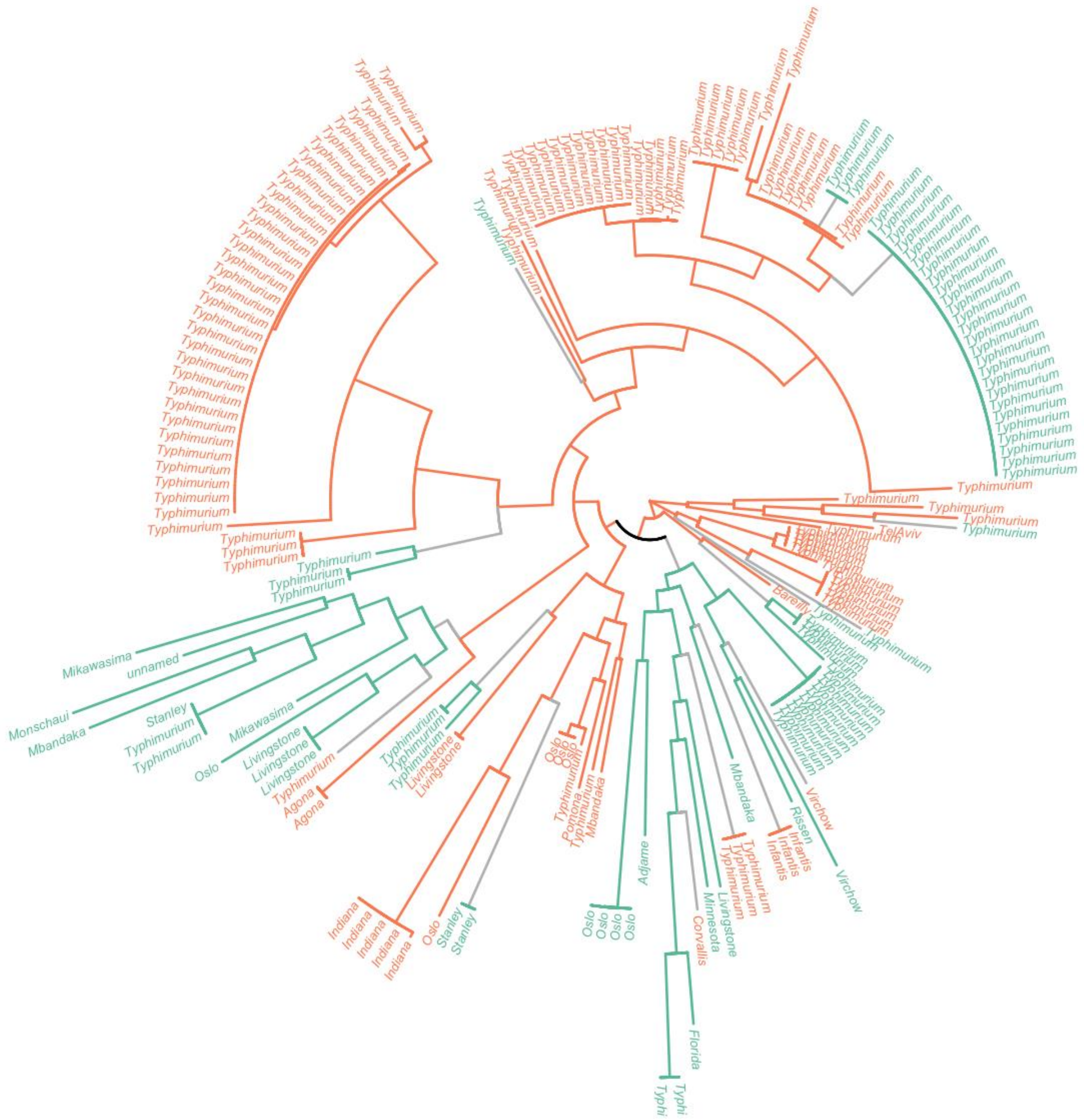

Fig. S1 Phylogenetic tree based on the 51,260bp alignment of 54 core genes of the 193 *repL*-positive phage-plasmid contigs (Fig. 1) coloured by plasmid replication type, as observed at the tips and inferred via ancestral state reconstruction along the branches (IncY = green, p0111 = orange), showing 17 changes in replication type state (IncY>p0111 (5/17) or p0111>IncY (12/17) = grey).

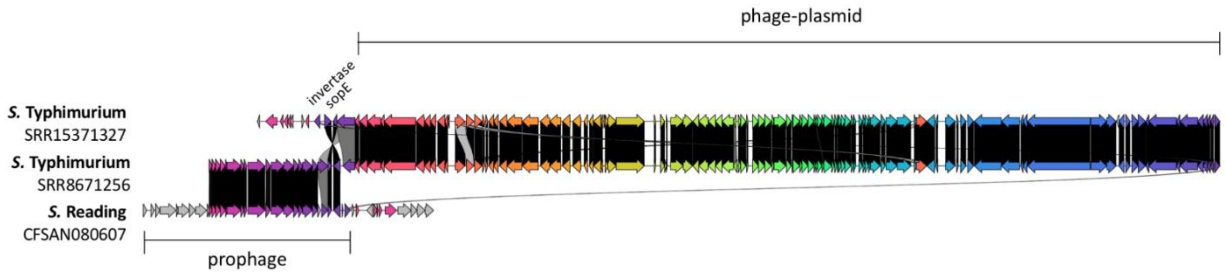

Fig. S2 Phage-plasmids in *S.* Typhimurium putatively integrated in chromosome at site of prophage.

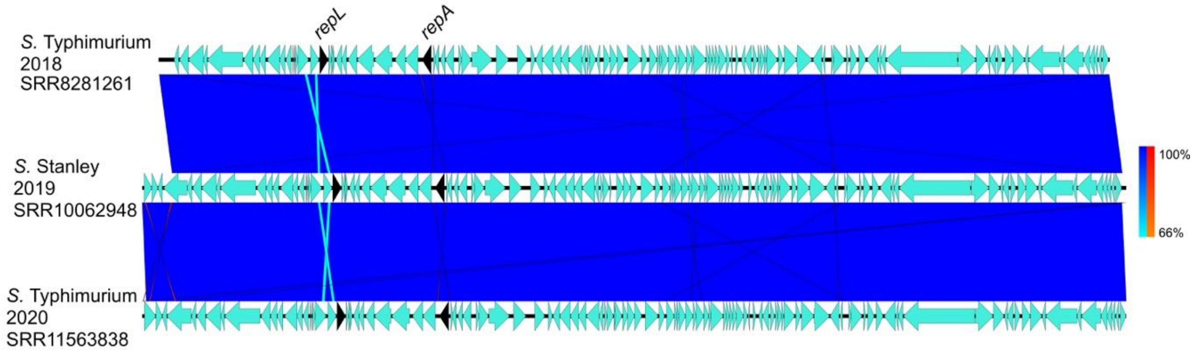

Fig. S3 Highly similar phage-plasmid sequences in different serovars: *S.* Stanley from 2019, and *S.* Typhimurium from 2018 & 2020. Arrows indicate gene direction and scale bars indicate level of similarity for forward (blue) and reverse (red) sequences.

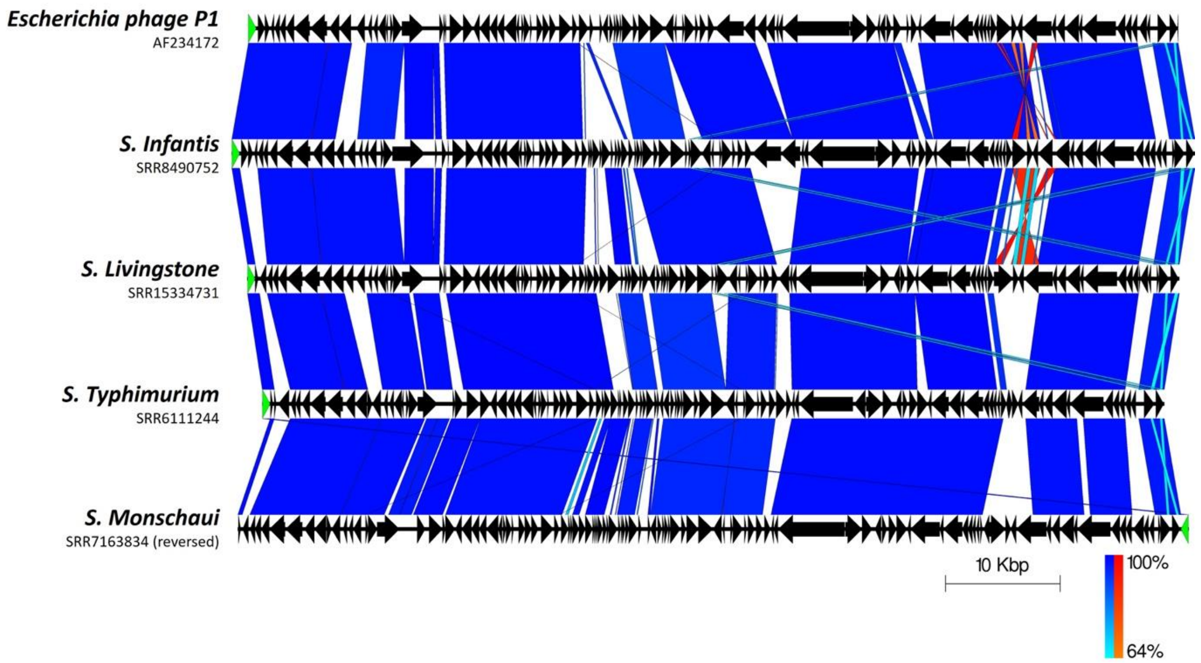

Fig. S4 Variation in selected phage-plasmids from multiple serovars (*S.* Infantis, *S.* Livingstone*, S.* Monschaui and *S.* Typhimurium) compared to P1 bacteriophage. Arrows indicate gene direction and scale bars indicate level of similarity for forward (blue) and reverse (red) sequences. The *repL* gene is highlighted in green.

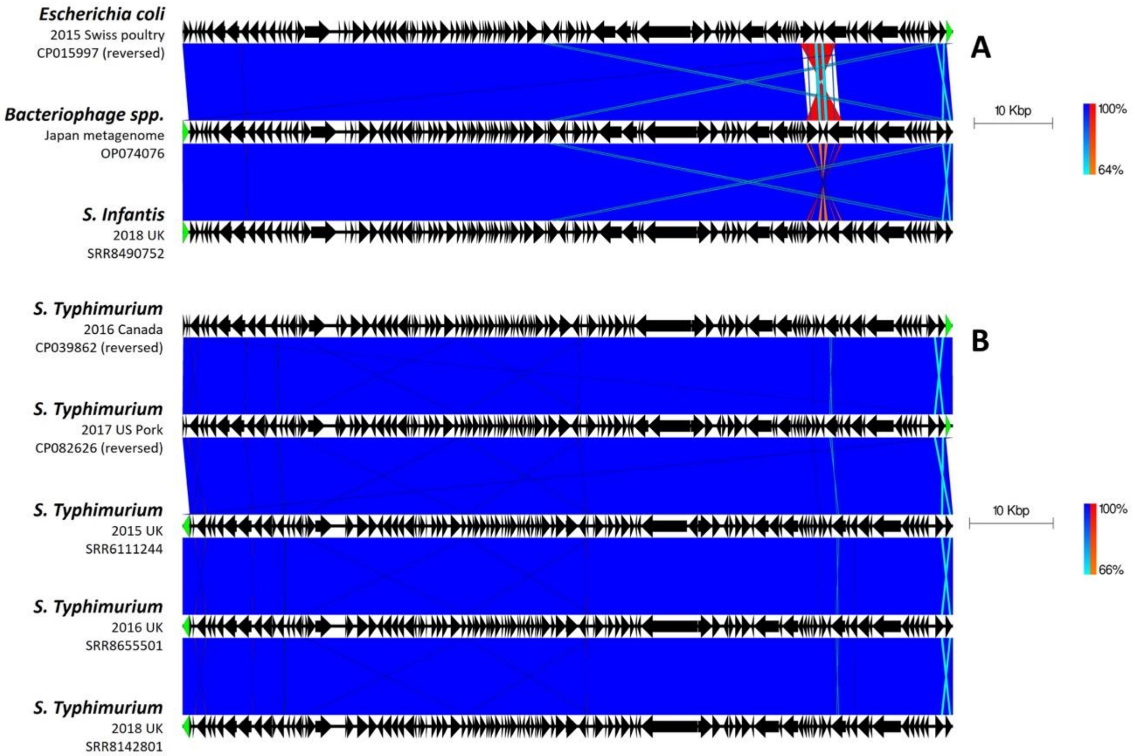

Fig.

Fig. S5 Highly similar phage-plasmid sequences from public databases showing conserved sequence across: **A)** different species; **B)** isolates of *S.* Typhimurium from different years and countries. Arrows indicate gene direction and scale bars indicate level of similarity for forward (blue) and reverse (red) sequences. The *repL* gene is highlighted in green.

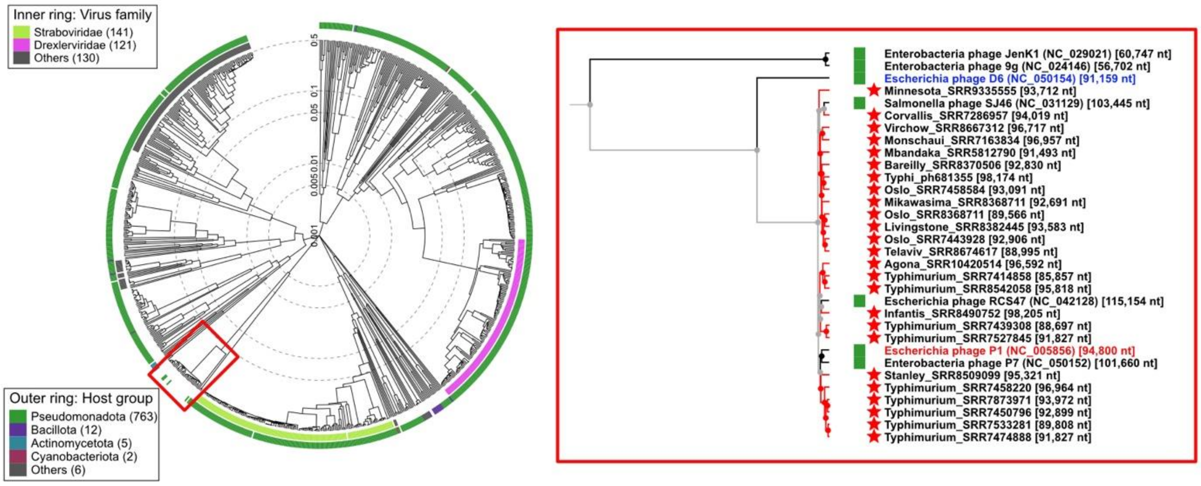

Fig. S6 Detailed output from ViPTree server showing taxonomic context of P1-like phage-plasmids. Left: proteomic tree based on tBLASTx sequence similarity of 825 prokaryotic dsDNA viruses, with location of the P1-like clade highlighted in red; Right: zoomed-in view of highlighted clade, showing 25 representative phage-plasmids from this study and their nearest relatives such as P1 (red), D6 (blue), P7 and SJ46.
